# Optical barcoding using polarisation sensitive plasmonic biosensors for the detection of self-assembled monolayers

**DOI:** 10.1038/s41598-022-16804-0

**Published:** 2022-07-29

**Authors:** Eugeniu Balaur, Catherine Sadatnajafi, Brian Abbey

**Affiliations:** 1 Department of Mathematical and Physical Sciences, School of Engineering, Computing and Mathematical Sciences, Bundoora, VIC 3086 Australia; 2 La Trobe Institute for Molecular Science, La Trobe University, Bundoora, VIC 3086 Australia

**Keywords:** Metamaterials, Nanophotonics and plasmonics

## Abstract

Periodic subwavelength apertures have the ability to passively detect variations in the dielectric properties of the local sample environment through modification of the plasmon resonances associated with these structures. The resulting resonance peak can effectively provide a ‘fingerprint’ indicative of the dielectric properties of the medium within the near-surface region. Here we report on the use of bimodal silver-based plasmonic colour filters for molecular sensing. Firstly, by exploring the optical output of these devices as a function of the incident polarisation for a range of different analytes of known refractive index, we were able to both maximise and quantify their sensitivity. We then apply this concept to the real-time monitoring of the formation of self-assembled monolayers based on detection of the optical output using a spectrometer. This highlights the potential for bimodal plasmonic devices to be able to dynamically monitor variations in the local environment down to the level of single molecules without the need for specific functionalisation or labelling. Advantages of using this technique include the ability for these devices to be miniaturised and to dynamically tailor their optical output permitting the analysis of very small sample volumes and maximise their dynamic range for a specific analyte.

## Introduction

The physical properties of nanoparticles and nanosized structures tend to be different from that of bulk materials. Recent developments in the field of nanofabrication such as the introduction of Electron Beam Lithography (EBL), Focused Ion Bram (FIB), and nanoimprinting have opened new avenues for creating bespoke structures at the nanoscale. These structures can in turn be used to generate and manipulate phenomena such as Surface Plasmon Resonance (SPR) and Extraordinary Optical Transmission (EOT). SPR describes the resonant interaction of light with free electrons, typically at a metal–dielectric interface^1^. The interaction between light and SPR mediated by sub-wavelength structures in thin films was investigated for the first time during the 1990s. In 1998 it was experimentally demonstrated by Ebbesen et al. that the transmission of light through an array of sub-wavelength apertures fabricated in thin metal films is greater than that predicted by classical optics. This was the first experimental demonstration of EOT and has since gone on to be exploited for wide range of different applications^2,3^. Two of the most common applications reported for devices exhibiting EOT phenomena are colour filtering and chemical sensing^4–7^. More recently, these types of devices have also been exploited for enhanced imaging^8,9^ and materials characterisation^10^.

The use of nanoapertures as the basis for chemical sensing offers a number of advantages including extremely high sensitivity to variations in the local Refractive Index (RI) and the ability to be miniaturised. As nanofabrication techniques have continued to advance, more complex apertures and structures are continuously being produced resulting in greater control over the EOT phenomenon for enhanced chemical sensing. In particular, plasmonic devices that alter their response depending on the polarization of incident light are of particular interest due to their multimodal functionality. Polarization sensitive plasmonic devices have been explored in the context of continuously tunable colour filtering^5–7^, wave-plates^11,12^ and multimodal chemical sensing^13^. Previous studies involving polarization mediated sensing have exploited asymmetric aperture designs such as arrays of cross-shaped holes in metallic thin films. These devices have shown bimodal plasmonic sensors can be used to differentiate analytes with varying RI via a colour change ‘by eye’ or via optical chemical barcoding, which provides a unique chemical ‘fingerprint’ in the measured spectra^13^.

Here, we extend the application of polarization sensitive plasmonic structures and optical barcoding to molecular sensing and demonstrate that such devices have the potential to detect the minuscule changes in the local RI associated with the attachment of different SAMs. We show that a unique molecular barcode or ‘fingerprint’ can be generated for SAMs of different length alkane chain but similar RI, and same length of the alkane chain but different RI. The approach of optical chemical barcoding using polarisation sensitive plasmonic colour filters was previously demonstrated using bulk chemicals of known RI. Here, we apply these concepts to the much more challenging application of molecular detection of SAMs. Key differences compared to bulk RI sensing include the fact that the SAM only extends a few nm into the exponentially decaying Z component of the electromagnetic field generated by the plasmonic device. This means that, unlike bulk chemicals, the molecules extend only a very small fraction of the skin depth. Here, we have specifically selected SAMs which probe the limits of sensitivity of this technique, for example, molecules which have the same RI but different chain length. A key hypothesis explored here is whether optical chemical barcoding allows the differentiation of molecular systems with very similar optical characteristics. The label-free detection of molecular assemblies of organic molecules represents unique challenges compared to bulk chemical sensing in terms of the interplay between molecular chain length and RI as well as the influence of molecular dynamics and variations in the contact angle. These issues are explored via the study of three different SAMs: 1-Hexa-decanethiol (HDT), 1-Octa-decanethiol (ODT), and 16-Mercaptohexadecanoic acid (16-MHDA). Further, we are able to interpret the results in terms of the physical properties of the molecular layers, such as film thickness and coverage. The implications of these findings are significant in the context of biomedical label-free molecular detection. We also demonstrate the possibility of using this approach for real-time molecular detection.

## Experimental details

**Figure 1:**
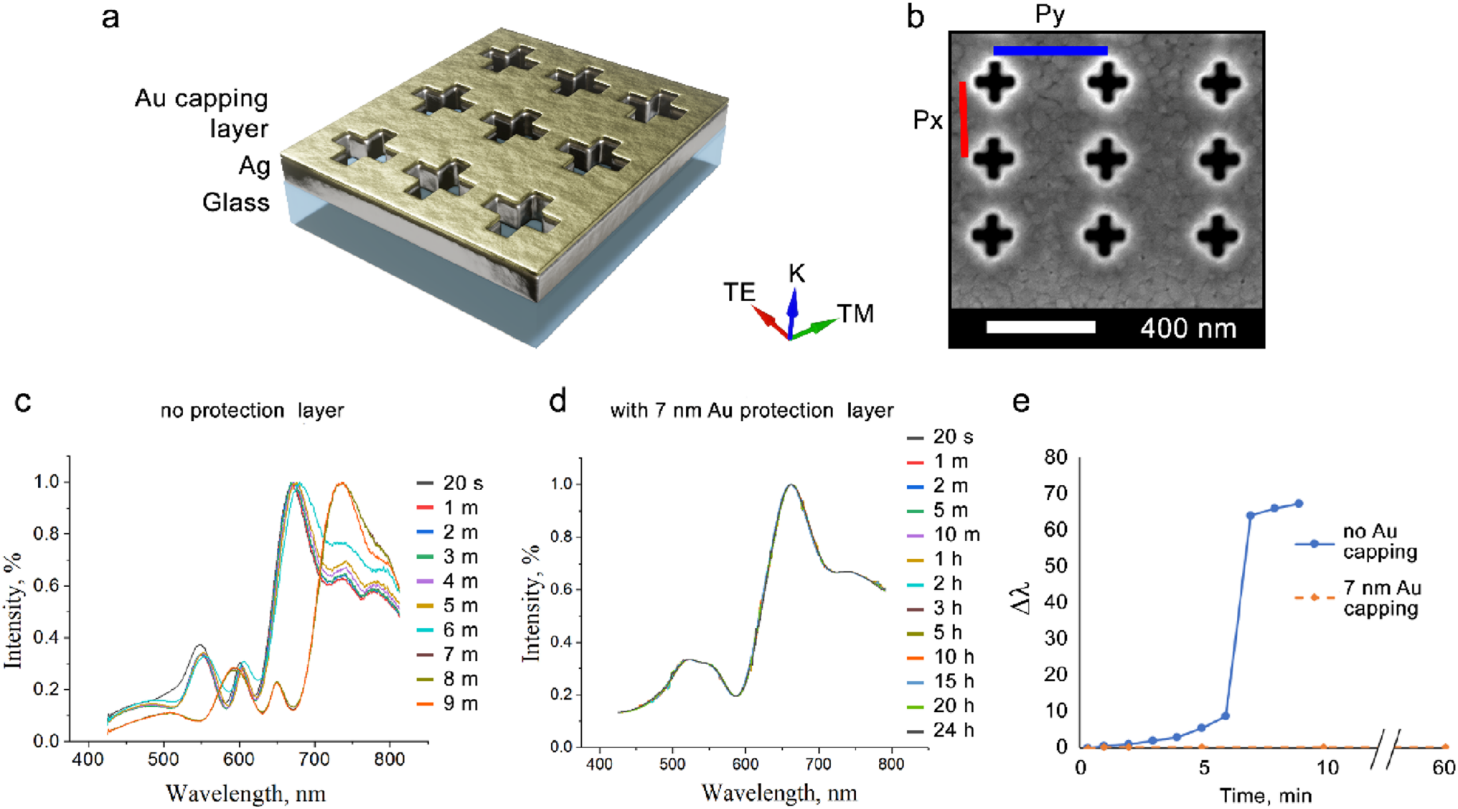
(**a**) A schematic device design and configuration. (**b**) SEM image of the cross-shaped apertures. *Px* (red line) and *Py* (blue line) indicate the array periodicities in the *X* and *Y* directions respectively. The width and the length of the arms of the cross-shaped apertures are 50 and 160 nm respectively. Transmission spectra for (**c**) a bare Ag plasmonic device and d) a device covered with a 7 nm Au film immersed in ethanol solution. (**e**) The corresponding shift in the main plasmon resonance peak. Note that at 7 min there is a large jump in the main transmission peak position for the bare Ag device and hence the measurements were halted for the bare plasmonic after 10 min. For the device protected by 7 nm Au the shift in plasmon resonance peak position was found to be < 0.5 nm after 60 min. The devices were also left in ethanol overnight for a total of 24 h and then re-measured and, even then, no peak shifts greater than 0.5 nm were observed.

Plasmonic devices were fabricated on quartz substrates, cleaned using sonication in acetone and isopropanol baths for 5 min, with each cleaning step followed by rinsing in deionised (DI) water. The substrates were further cleaned in hot piranha solution for 15 min, rinsed with copious amounts of DI water and dried with a N_2_ gaseous stream. Electron-beam evaporation (Intlvac Nanochrome II) was used to deposit films on the pre-cleaned quartz substrates. Immediately prior to deposition, the quartz substrates underwent a final clean using a broad beam Ar ion source for a total of 5 min. A 6 nm thick Ge adhesion layer was then deposited onto the quartz substrate at a deposition rate of 0.3 (Å/s). Silver films of 150 nm thicknesses were then deposited at a rate of 0.5 (Å/s). After the preparation of the silver films, periodic arrays of nanoscale cross-shaped apertures with periodicities of *P*_*x*_: 400 and *P*_*y*_: 450 nm in the *x* and *y* directions (see Fig. 1) were milled using the Focused Ion Beam (FIB) technique (FEI Helios NanoLab 600 FIB-SEM). The FIB system consisted of two integrated beams, an electron beam for scanning electron microscopy (SEM) and a Ga ion beam for milling. The milling of the structures was performed using a 30 kV ion beam and a 9.7 pA current. Capping Au films were deposited at a rate of 0.5 (Å/s). Fresh samples were immersed in 1 mM SAM (MHDA, HDT or ODT) ethanolic solutions for 24 h. Table 1 summarizes the SAM parameters used in this study. After immersion in the SAM solution, the samples were cleaned using pure ethanol followed by a wash in DI water and finally dried using N_2_ gas. The optical performance of the fabricated plasmonic devices was evaluated using a combination of a wide-field optical microscopy (Nikon Instrument A1Rsi + Ti-E) and transmission spectroscopy (Ocean Optics QEPro). The transmission spectra were collected with a 50 ms exposure time and 10 exposures per frame. Optical images and transmission spectra were collected using linearly polarized light at three different polarizations: Transverse Electric (TE), 45°, and Transverse Magnetic (TM).

**Table 1:** The molecular formula, refractive index, permittivity, and chain length of the SAMs used in this study. Values are taken from Refs^14–17^.

SAM name	1-Hexa-decanethiol (HDT)	1-Octa-decanethiol (ODT)	16-Mercaptohexadecanoic acid (16-MHDA)
Molecular formula	C_16_H_33_SH	C_18_H_37_SH	C_16_H_32_O_2_S
Refractive index (*n*)	1.462	1.483	1.765
Permittivity (*ε*_*SAM*_)	2.14	2.20	3.16
Chain Length (nm)	2.0	2.8	1.9

## Results and discussion

### Device passivation and performance

Due to its electronic properties, Ag is a prime candidate as a material of choice for plasmonic devices in the visible range, however, it suffers from rapid degradation when left unprotected, particularly in wet environments. For example, a noticeable degradation of Ag is observed in the ethanolic solutions used to deliver SAMs to the surface of plasmonic devices^14^. Specifically, selective oxidation of ethanol by Ag films leads to the formation of silver aggregates and recrystallization^14,18–20^. Since the surface of the plasmonic device needs both to protect the active Ag film and to provide a facile surface for the molecules to attach to, Au was selected as a suitable capping layer. Whilst Au is both chemically inert and can be easily deposited, its performance within the optical regime for plasmonic sensing is inferior to Ag. It is well-established in the literature that within the visible wavelength regime silver-based plasmonic sensors achieve superior sensitivity compared to gold-based plasmonic sensors for an identical device design. Typically, the sensitivity of gold-based plasmonics only becomes comparable to silver-based plasmonics at longer wavelengths (> 650 nm) where sensitivities of up to 400 nm/RIU have been reported^21^. The reasons for this can be understood in terms of the underlying electronic properties of these two materials. The disadvantage of silver-based plasmonics is that they require additional protection from the environment, in the present paper this is achieved by applying an additional inert capping layer which is a few nm thick. Although this adds additional complexity to the device fabrication it enables us to achieve superior performance in the visible range whilst maintaining biocompatibility. In previous work sensitivities of up to 435 nm/RIU were demonstrated using silver-based plasmonic sensors for wavelengths between 400 and 650 nm. Comparable sensitivities for gold-based plasmonic sensors within this wavelength range have been reported as just 125 nm/RIU^22^. We note that RI sensitivities for gold-based plasmonically active fiber tips have been reported to be as high as ∼ 533 nm/RIU when specifically comparing methanol (RI = 1.326) and ethanol (RI = 1.359)^23^. Therefore, it is widely acknowledged in the literature that silver is the preferred material over gold where high sensitivity is the priority. Hence, a very thin 7 nm layer of Au was used here to enable the formation of a continuous capping layer free from pores or defects, whilst only having minimal impact on the performance of the Ag plasmonic devices. We note that 7 nm is much less than the typical skin depth of these devices in ethanol which is ~ 100 nm at λ: 550 nm^19,24,25^, allowing for a substantial sensing volume to be maintained above the device surface. To test the stability of the plasmonic devices in-situ, with and without the Au protective layer, freshly prepared devices were immersed in ethanol and their transmission spectra periodically measured (Fig. 1).

For the bare Ag plasmonic device, a significant redshift of > 60 nm occurs after 7 min immersion in ethanol; this confirms the expected degradation of Ag which happens very rapidly due to oxidation of the surface. Given that the formation of a single SAM layer will only induce a relatively small shift in the resonance peak position, it is clear that the bare plasmonic is unsuitable for molecular sensing in-situ. By contrast, just 7 nm of gold, deposited on top of the devices was found to lead to a dramatic improvement in the chemical resistance of the plasmonic device. Even after 24 h immersion in ethanol there was a < 0.5 nm shift recorded for the primary resonance peak which is at the limit of detection for the spectrometer.

**Figure 2:**
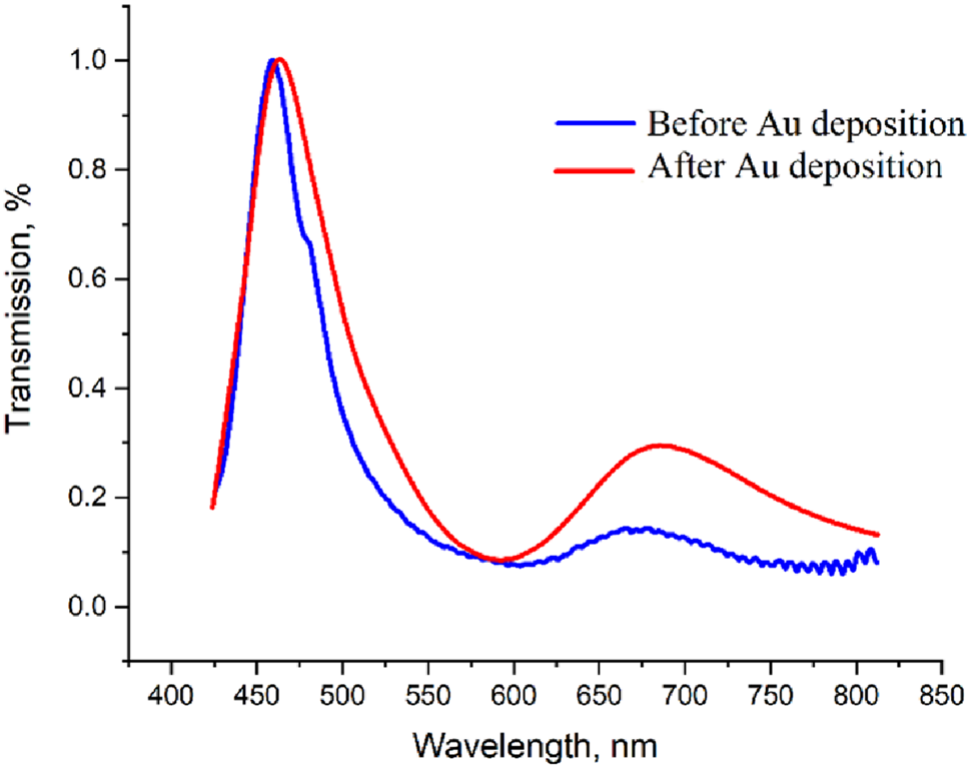
Normalised transmission spectra of a plasmonic device measured in air before and after capping with 10 nm of Au.

Although a thin Au capping layer was found to provide sufficient protection in the preliminary ethanol trials, this was increased to 10 nm for the final plasmonic devices used for the SAM measurements to provide a further margin for error in case of any small variations in the porosity or coverage of the thin film deposition. Figure 2 compares the results for the same plasmonic device before and after capping with 10 nm of Au. As expected, the thin Au layer only has a minimal influence on the main plasmon resonance peak resulting in a shift of just 4 nm. This shift partially occurs due to the fact that Au exhibits its own plasmonic modes in the visible range^25^.

Having established a reliable means for fabricating plasmonic devices for molecular sensing, a set of model SAMs against which to benchmark the performance of these devices was investigated. A summary of the optoelectronic properties of the SAMs used for this study is presented in Table 1. The specific SAMs chosen include HDT and ODT, which contain different numbers of carbon atoms in the molecular chain, and yet have a very similar RI. By contrast HDT and 16-MHDA have the same number of carbon atoms but very different RI properties.

### Theoretical calculation of the device sensitivity for SAM detection

The decay of the *Z* component of electromagnetic field in plasmonic devices is characterized by ‘skin depth’, $\delta z$. It is defined as the distance over which the *Ez* component of the electric field associated with the SPP wave decays to *1/e* of its initial value. A value for the skin depth can be determined by considering the dielectric constant for the metal and SAM, $\varepsilon_{m}$ and $\varepsilon_{SAM}$ respectively assuming a quasi-infinite layer thickness:

1 $$\delta z = \frac{1}{{k_{0} }}\left| {\frac{{\varepsilon_{m} + \varepsilon_{SAM} }}{{\varepsilon_{SAM}^{2} }}} \right|^{\frac{1}{2}}$$

**Figure 3:**
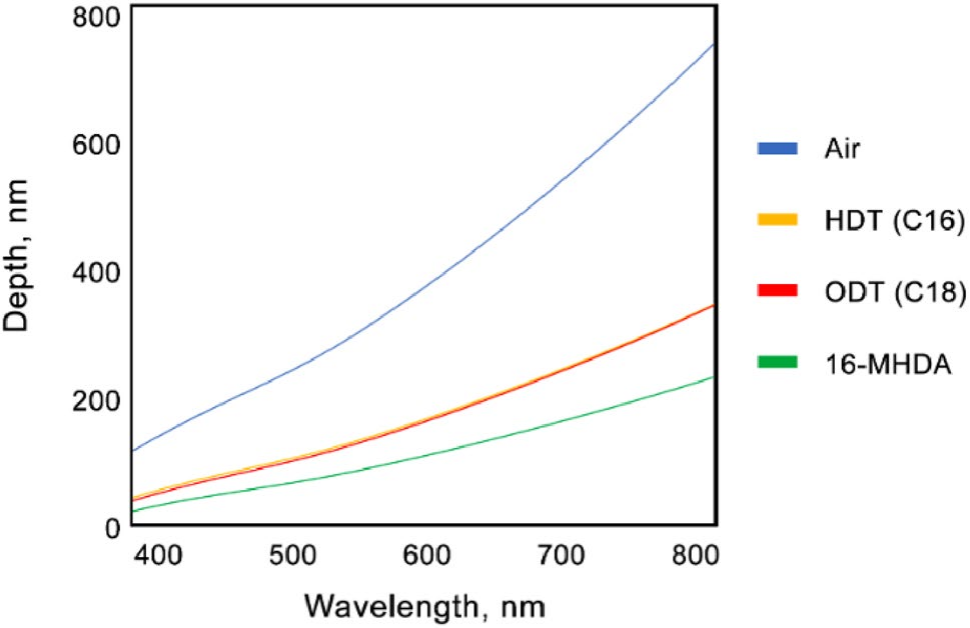
Skin depth calculated for air, HDT, ODT and 16-MHDA using Eq. (1).

The skin depth for HDT, ODT and 16-MHDA at an incident wavelength of λ = 550 nm was found to be: 117, 114 and 76 nm respectively. Figure 3 presents the calculated skin depth across the whole visible range for a dielectric layer which is just air as well as for the three SAMs used in this study.

Figure 3 shows that in terms of the skin depth, HDT and ODT, are quite similar in spite of their differences in terms of chain lengths. Whereas the skin depth for 16-MHDA is quite different to that of HDT and ODT by virtue of its distinct RI properties despite having the same chain length.

**Figure 4:**
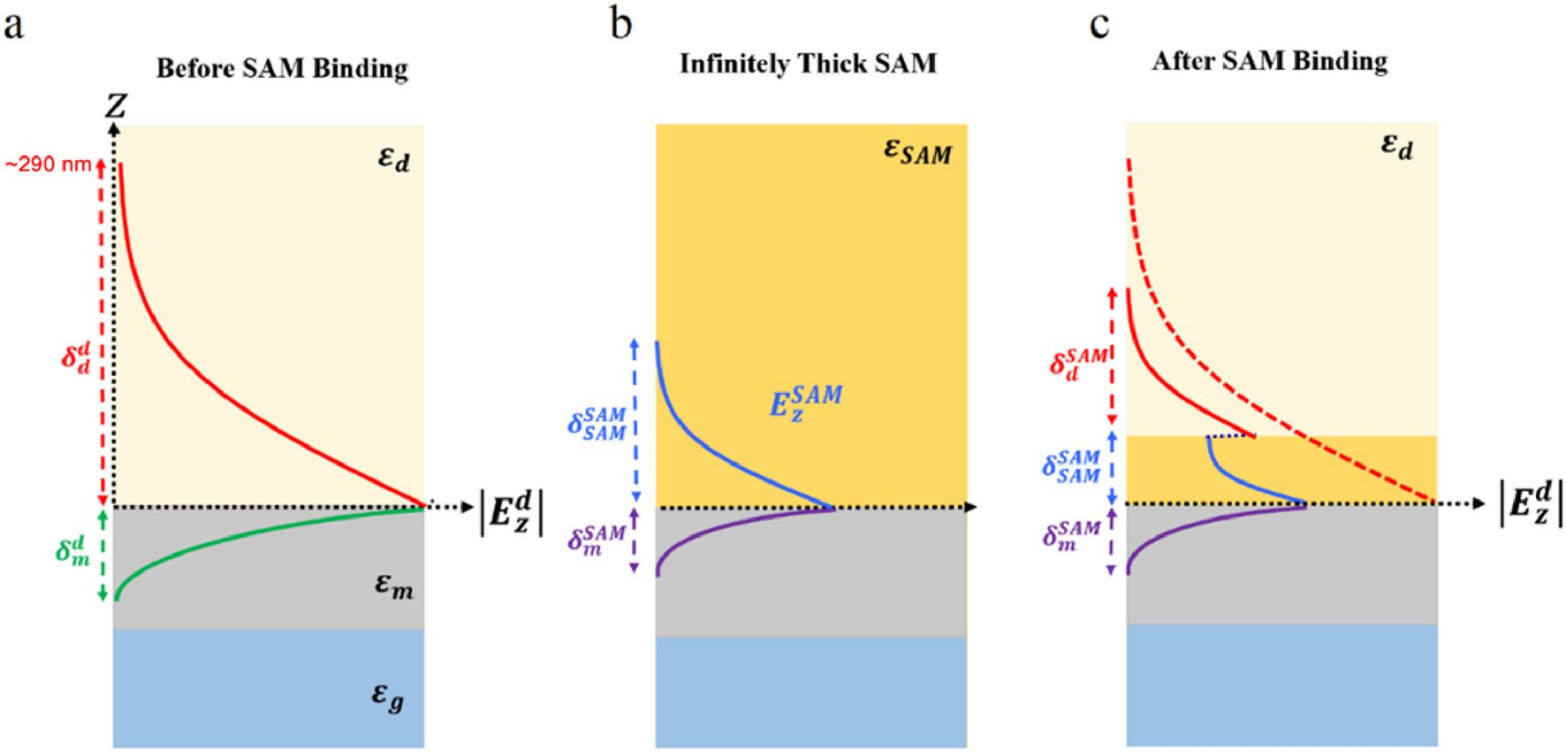
Schematic of the decay of the *E*_*z*_ component associated with the SPP EM field for (**a**) free-space propagation, (**b**) in a quasi-infinitely thick SAM and (**c**) in a single SAM just one molecule thick. The dotted arrows denote the extent of the field in metal (${\varvec{\delta}}_{{\varvec{m}}}^{{\varvec{d}}} \user2{ }$ and ${\varvec{\delta}}_{{\varvec{m}}}^{{{\varvec{SAM}}}}$), air (${\varvec{\delta}}_{{\varvec{d}}}^{{\varvec{d}}} \user2{ }$ and ${\varvec{\delta}}_{{\varvec{d}}}^{{{\varvec{SAM}}}}$), and in the SAM (${\varvec{\delta}}_{{{\varvec{SAM}}}}^{{{\varvec{dSAM}}}}$). The thickness of the metal layer is ~ 150 nm.

Historically, detection of SAM formation via conventional SPR approaches has proven to be quite challenging. As the differences in the skin depth demonstrate, the decay of the electromagnetic wave associated with the SPPs can be highly dependent on the local RI. Figure 4 presents a schematic of the *E*_*z*_ component of the electric field arising from the propagation of SPPs in three different media: air, a quasi-infinitely thick SAM, and (after formation) a SAM which is just one molecule thick.

The maximum skin depth for the plasmonic devices investigated here is approximately 290 nm at λ = 600 nm when the SPP electromagnetic field propagates in air (Fig. 4). However, as the permittivity increases the corresponding skin depth gradually decreases. In the case of a single SAM layer, the exponential decay curve initially matches that of the quasi-infinitely thick SAM (Fig. 4b). However, once the electric field extends beyond the single molecule layer, the rate of exponential decay instead matches that of air (Fig. 4c). This results in a discrete step-change in the decay rate at the upper SAM-air boundary.

For the majority of practical sensing applications, the wavelength (or angle) shift is recorded as a function of the change in the RI of the medium being investigated. The relationship between these parameters is known as the sensitivity factor and is given by:

2 $$S = \frac{{\Delta \lambda_{SPP} }}{\Delta RI}$$

were the position of the resonant peaks $\Delta \lambda_{SPP}$ can be determined from the dispersion equation for resonant peaks associated with different Bloch modes of the SPPs at normal incidence^26^:

3 $$\lambda_{SPP} \cong \frac{P}{{\sqrt {i^{2} + j^{2} } }}\sqrt {\frac{{\varepsilon_{m} \varepsilon_{d} }}{{\varepsilon_{m} + \varepsilon_{d} }}}$$

where *P* is the array periodicity and *i* and *j* are integers corresponding to the scattering orders from the aperture array.

**Figure 5:**
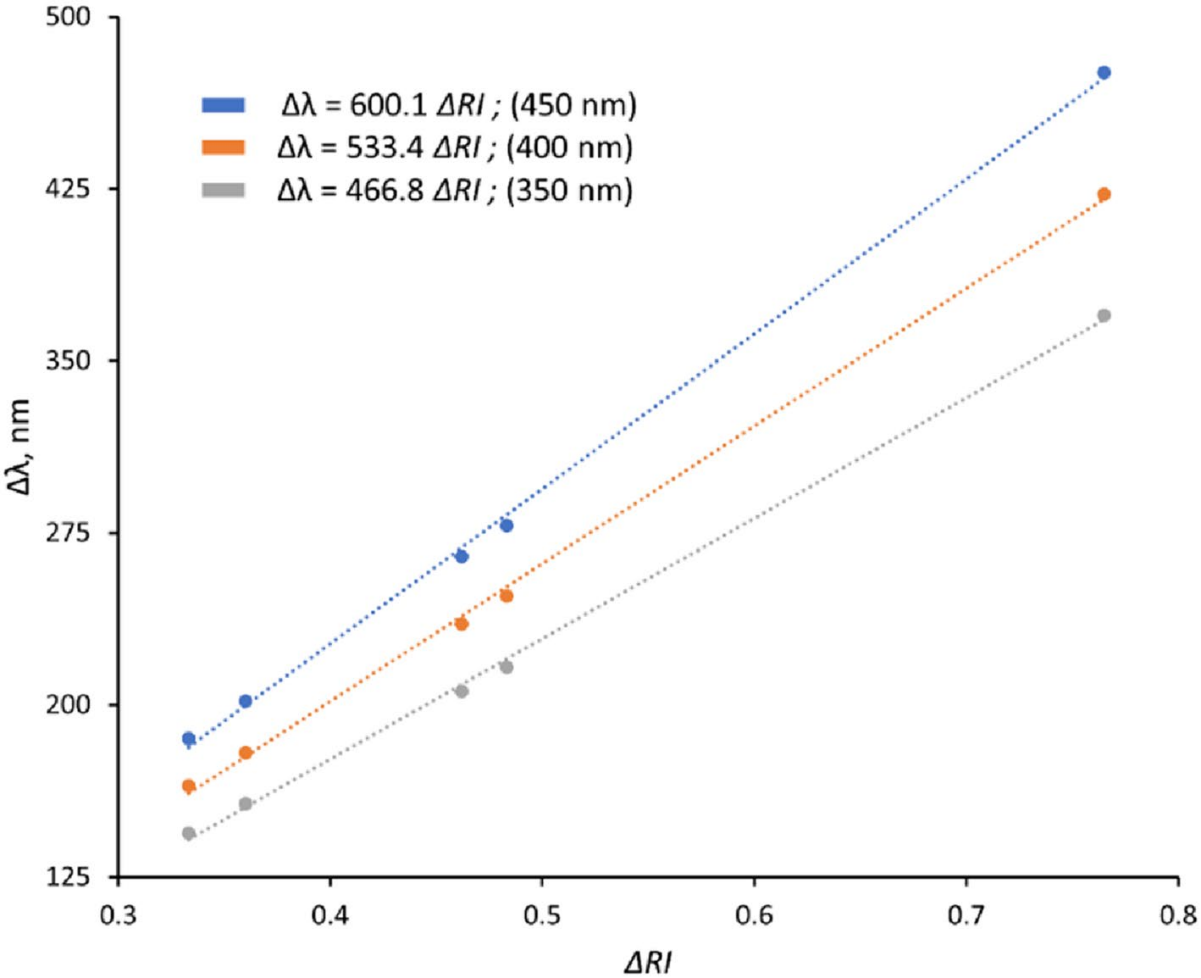
The calculated sensitivity factor (Eq. 2) for the plasmonic devices used for SAM detection corresponding to three different pattern periodicities: 350, 400 and 450 nm.

The *S* factor gives an indication of the expected wavelength (or angle) shift as a function of the change in RI. Using Eq. (2) the expected sensitivity for three different device periodicities used for these measurements (350, 400, and 450 nm) was calculated and is summarised in Fig. 5.

In the case of a finite SAM film with a thickness in the range of the skin depth, the SPR response, *R*, is given by:

4 $$R = S (n_{SAM} - n_{d} )[1 - \exp \left( {\frac{{ - 2t_{SAM}^{th} }}{\delta z}} \right)]$$

where *n*_*SAM*_ and *n*_*d*_ are the RI of the SAM and the dielectric medium immediately adjacent to the SAM respectively; *t*_*SAM*_ is the SAM thickness and *δz* is the skin depth in a quasi-infinite SAM. In the case of a highly ordered monolayer, ${t}_{SAM}$ is very similar to the thiol molecule length, therefore, the Eq. (3) can be used to estimate the theoretical expected resonant shift for a given SAM. Likewise, from the experimental evaluation of the resonant spectral shift, $t_{SAM}$ can be calculated using the following derived relation based on Eq. (4):

5 $$t_{SAM}^{exp} = \left( {\frac{{\delta_{z} }}{2}} \right)\left( {\frac{{R_{exp} }}{{S\left( {n_{SAM} - n_{d} } \right)}}} \right)$$

By comparing the experimental to the theoretically predicted SAM film thickness it is thus possible to estimate the total SAM coverage using:

6 $$C = \frac{{t_{SAM}^{exp} }}{{t_{SAM}^{th} }} \times 100\%$$

Equations (4–6) were used to generate theoretical predictions against which to benchmark the experimental SAM data. The results are summarised in “SAM refractive index and coverage” Section.

### SAM refractive index and coverage

**Figure 6:**
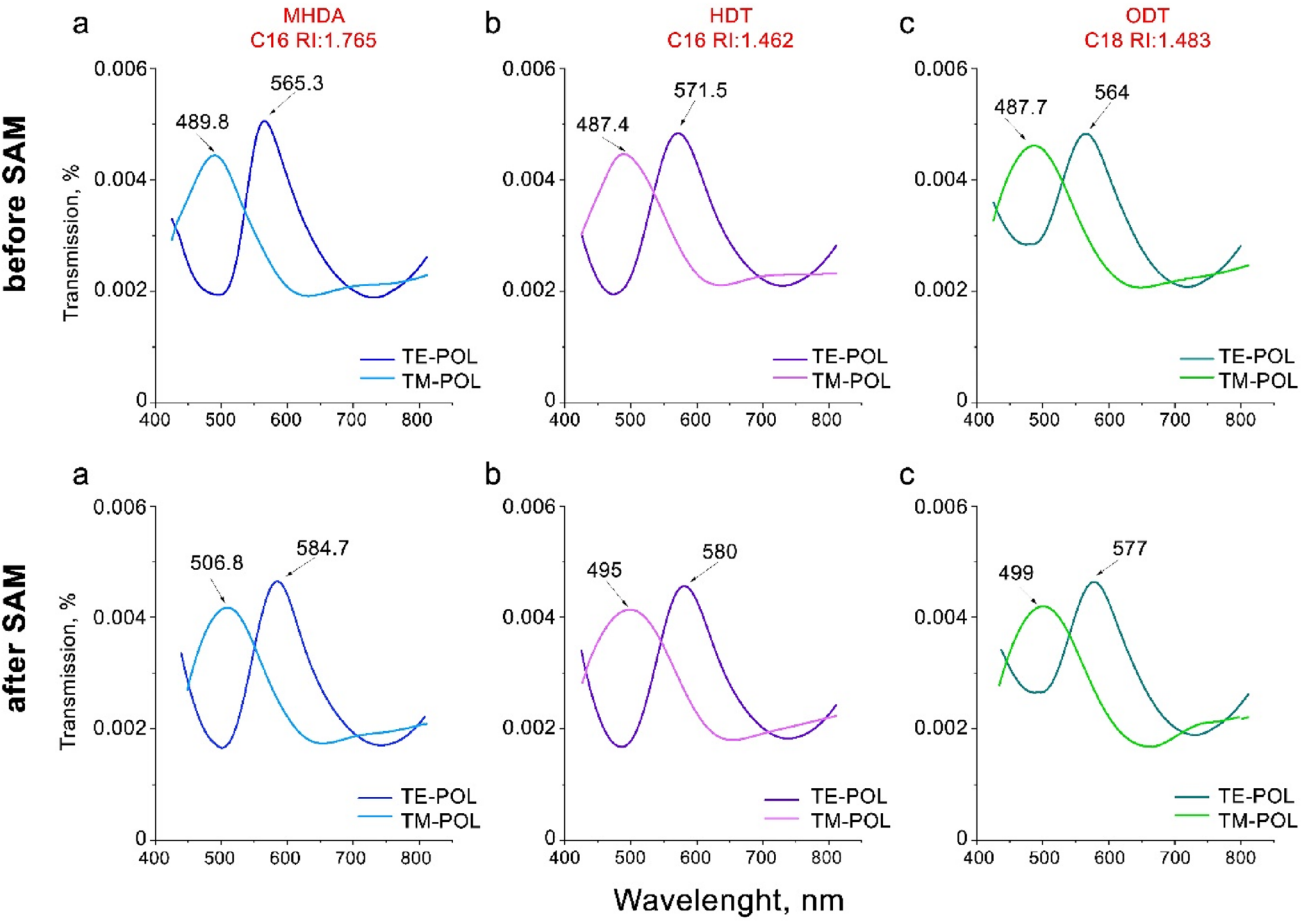
Transmission spectra taken in air for three devices: top row—before and bottom row—after deposition of MHDA, HDT and ODT SAMs respectively.

Polarization-sensitive devices with cross-shaped apertures fabricated with periodicities of ${P}_{x}$: 450 and ${P}_{y}$: 400 nm were used to study the formation of three SAMs. As discussed in the “Theoretical calculation of the device sensitivity for SAM detection” section, performing measurements at two different polarizations results in two independent sets of transmission data. This is because each polarization mode (TE or TM) has a unique spectrum associated with a particular periodicity within the bimodal devices. Similar trends in the spectrum for both polarizations are expected for a particular SAM, but we can use the mode corresponding to the greatest dynamic range in order to extract the most accurate sample properties. A transmission spectrum was first collected in air prior to immersion in 1 mM SAM (MHDA, HDT or ODT) ethanolic solutions for 24 h. This amount of time in the SAM is more than sufficient to form a complete layer. A comparison of the measured transmission spectra before and after SAM deposition is shown in Fig. 6 for both TE and TM incident polarisation modes.

As expected, the spectra in Fig. 6 reveal larger redshifts for the TE polarization mode, which is associated with the 450 nm periodicity. In accordance with the Eq. (3), a larger array will induce a larger wavelength shift associated with a particular dielectric constant. In addition, we observe that the redshift is larger for SAMs with the same chain length, but different RI, compared to SAMs which have the same RI but different chain length. These results are consistent with the predicted skin depth calculations (see Fig. 3).

The red shift of the primary plasmon resonance peak provides a well-established means of tracking the formation of the SAM on the plasmonic device surface^27^. Using Eq. (5), the experimental SAM thickness can be determined from this peak shift. Once the SAM thickness, ${t}_{SAM}^{exp}$ is extracted, the total SAM coverage within the measurement area can be estimated by using Eq. (6). A summary of the theoretically predicted and experimental results for the SPR response, as well as the experimental data for the SAM thickness and coverage is provided in Table 2.

**Table 2:** Theoretical and experimental comparison of the SPR response (Eq. 4), SAM thickness (Eq. 5) and coverage (Eq. 6) for TE and TM polarisation.

SAM type	*R*_*theoretical*_ (nm/RIU)		*R*_*experimental*_ (nm/RIU) ± 0.2		$t_{SAM}^{th}$(nm) ± 0.15	$t_{SAM}^{\exp }$(nm) ± 0.2		$C_{SAM} \left( \% \right)$	
	TE	TM	TE	TM		TE	TM	TE (%)	TM (%)
MHDA	22.4	19.9	19.4	17	1.9	1.61	1.58	85	83
HDT	9.3	8.3	8.5	7.5	2.0	1.79	1.78	90	90
ODT	13.9	12.3	13	11.3	2.8	2.56	2.50	91	90

Note that the theoretical SAM thickness, ${{\varvec{t}}}_{{\varvec{S}}{\varvec{A}}{\varvec{M}}}^{{\varvec{t}}{\varvec{h}}}$, is given by the chain length (see Table 1).

In general, long-chained SAMs (> 18 °C) such as ODT tend to form denser films^28^. This is supported by the experimental data based on the shift in the primary resonance peak position which shows a higher degree of coverage for the ODT then for the MHDA. The consistency in trends for *R* and the reasonable match between the theoretical and experimental values for *R* and for ${t}_{SAM}$ provide confidence in the ability of bimodal Ag plasmonic devices to detect SAM adhesion. Having established that the shift of the primary resonance peak for our devices provides a means of detecting and characterising SAMs, we now explore whether optical barcoding can be used to differentiate between the three different SAMs.

### Using the isosbestic points for optical barcoding of SAMs

In “SAM refractive index and coverage” section we determined that the position of the resonance peak can be quantitatively analysed in order to extract the SAM thickness and coverage. Next, the goal was to explore how sensitive optical barcoding is to the deposition of the SAM. The optical barcoding approach we have developed exploits features in the spectrum known as ‘isosbestic points’. These are specific well-defined wavelengths at which the optical response of the plasmonic device is invariant with respect to the incident polarisation, for additional information regarding the isosbestic points, the reader is directed to Ref^13^. Previous work investigated the use of isosbestic points for chemical sensing of liquids where the analyte extended beyond the skin depth and was delivered to the plasmonic device using microfluidics^13^. Here we explore this concept in terms of ‘fingerprinting’ single molecule layers which, since the deposition of SAMs is an irreversible process, requires a fresh (albeit nominally identical) device to be used for each SAM. The spectral data collected from the three plasmonic devices used for this study prior to deposition and following 24 h immersion in the SAM is presented in Fig. 6. Whilst the data ‘before SAM’ spectra are very similar for the three devices, small nanoscale variations in the fabrication of the devices using FIB results in the primary resonance peak position shifting position by up to 6.2 nm. Hence, in the case of optical barcoding for molecular sensing we analyse the relative changes ‘before SAM’ and ‘after SAM’ for each of the three plasmonic devices individually, rather than compare absolute values between different devices. By analysing the relative (rather than absolute) differences, these small deviations in device characteristics no longer have an impact in terms of tracking SAM coverage.

**Figure 7:**
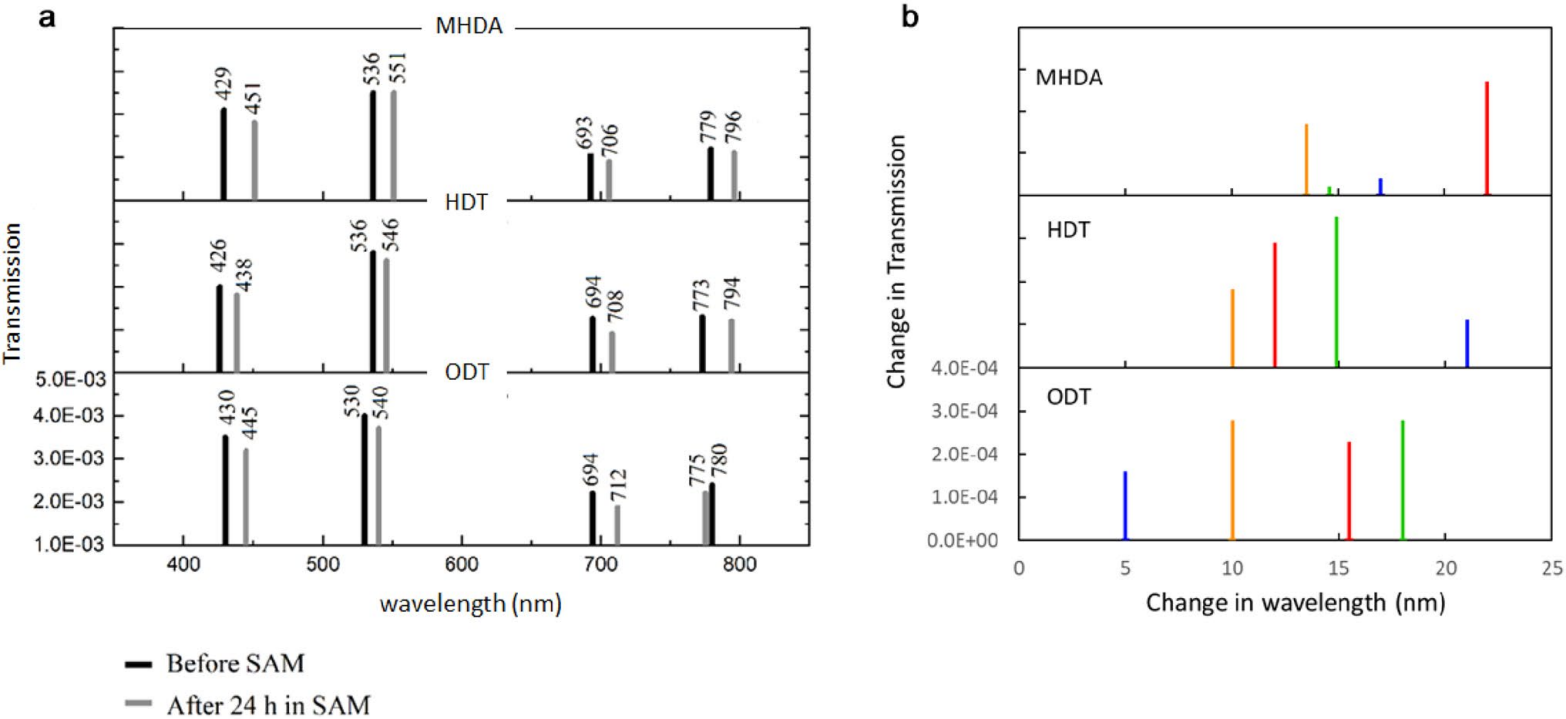
Optical barcodes for SAMs (**a**) ‘before SAM’ (vertical black lines) and ‘after SAM’ (vertical grey lines) and (**b**) position and transmission of isosbestic points for MHDA, HDT, and ODT.

Figure 7 presents a summary of the position and intensities of the isosbestic points (or ‘optical barcodes’) prior to SAM deposition (darker bars) and after 24 h immersed in the SAM (lighter bars). In all but one case we note that the shift in position of the isosbestic point is far greater than the differences in the absolute values of the isosbestic points due to slight variations in nanofabrication between the three different devices.

Figure 7a shows that there are four isosbestic points present in these spectra. Analysis of the data collected from each of the three devices used to detect the presence of MHDA, HDT, and ODT reveals that relative to the initial spectra the isosbestic points are clearly different for each of the three SAMs, both in terms of their position and their intensity. We note that these differences cannot be accounted for by the small changes in the device output as a result of variations in nanofabrication of specific devices. In summary, the magnitude of the changes observed in these optical barcodes indicate a clear distinction between the three different SAMs measured. For comparison the change in position of the main resonant peak positions measured in “SAM refractive index and coverage” section were Δ*λ* (TM/TE) = (17.0/19.4), (7.6/8.5), and (11.3/13.0) nm for MHDA, HDT, and ODT respectively. These changes were attributed to the RI of the particular SAM and local changes in SAM coverage. We emphasise here, that the use of optical barcoding only applies to the detection of the presence of the SAM, this approach is not used to quantify the SAM coverage and thickness as it would require a priori calibration. For the extraction of the quantitative SAM properties, we used the position of the primary resonance peak, using the mode which provided the largest dynamic range. Hence, whilst the shift in resonant peak position can be used to obtain a quantitative interpretation of parameters such as the SAM coverage, the use of isosbestic points for molecular detection appears extremely promising in terms of sensitivity and discrimination. In particular, with further improvements in the consistency of nanofabrication optical barcoding could enable specific molecules to be distinguished as they attach to the surface, without requiring any specific surface functionalisation.

## Conclusion

Passive optical detection of variations in the local dielectric properties due to formation of thiol SAMs was demonstrated by using bimodal periodic subwavelength metallic apertures fabricated in thin Ag films. It was shown that the position of the resonant peaks can be used not only to distinguish different types of SAMs, but also for a direct quantification of the SAM coverage. It was observed that the long-chained SAMs tended to form denser films, in line with the published literature^29^. Whilst this method gave clear results following complete formation of the SAM, we note that it was sometimes difficult to reliably detect the very earliest stages (i.e. < 60 min) of SAM adhesion. To overcome this, the use of optical barcoding was explored in the context of molecular sensing. Whilst the results appear very promising, future work will address issues around the consistency of device fabrication through the development of alternative nanofabrication protocols. In addition, it may be possible in the future to further investigate the performance of bimodal plasmonics in terms of molecular sensing by building up, layer-by-layer, multiple layers of the same molecule. It is anticipated that this will enable an unambiguous comparison of the dynamics between different SAMs leading to in-situ monitoring of the dynamics of molecular adhesion and ordering.

## Data Availability

Most of the data generated or analysed during this study are included in this published article. Some data that support the findings of this study are available from the authors upon reasonable request (E. Balaur, e.balaur@latrobe.edu.au).
